# 70-year-old Woman with Chest Tightness and Shortness of Breath

**DOI:** 10.5811/cpcem.47061

**Published:** 2025-12-07

**Authors:** Robert E Dunn, Brianna Klucher, Laura J Bontempo, J David Gatz

**Affiliations:** * University of Maryland Medical Center, Department of Emergency Medicine, Baltimore, MD; † University of Maryland School of Medicine, Department of Emergency Medicine, Baltimore, MD

## Abstract

Chest tightness and shortness of breath are relatively common reasons for presentation to the emergency department (ED), often triggering protocolized workups and dispositions. A good history, however, can reveal additional elements that may dramatically alter the differential diagnosis and management. A 70-year-old woman presented to the ED complaining of subacute chest tightness with dyspnea on exertion. This case offers a thoughtful analysis of how to integrate key findings within a patient’s history, exam, and workup in the ED. The surprising final diagnosis and case outcome are then revealed.

## CASE PRESENTATION (DR. DUNN)

A 70-year-old woman was brought into the emergency department (ED) by her family for intermittent chest tightness over the prior one month. The chest discomfort was non-radiating, but worsened with exertion, and was associated with fatigue and dyspnea on exertion. Her daughter reported that the patient’s heart rate had been bradycardic to 40 beats per minute (bpm) for a few months. On a review of systems, the patient endorsed decreased appetite, generalized abdominal pain, diarrhea, bilateral leg and back pain for months, and generalized weakness and fatigue.

Her medical history was notable for heart failure with reduced ejection fraction (HFrEF) of 20–25%, non-ischemic cardiomyopathy, coronary artery disease, atrial fibrillation, and dyslipidemia. Her home medications included atorvastatin, dabigatran, empagliflozin, famotidine, furosemide, metoprolol succinate, and sacubitril/valsartan. Her son noted the patient had been non-compliant with dabigatran for an unknown amount of time. Family history was significant for cardiomyopathy. The patient lived with her daughter, was a former smoker, and denied using drugs or alcohol. She had moved from Venezuela one year earlier.

On initial presentation, the patient’s vital signs were as follows: temperature, 36.6 ˚C; heart rate, 40 bpm; blood pressure, 103/54 millimeters of mercury; respiratory rate, 14 breaths per minute; and oxygen saturation, 99% on room air. Her estimated body mass index was 20 kilograms (k) per square meter (reference range 18.5–24.9 k). On examination, the patient was well appearing and not in acute distress. Her head was normocephalic. Her neck had full range of motion and no jugular venous distension. Her heart had a regular but bradycardic rhythm. A soft gallop was heard on auscultation, but no other abnormal heart sounds were heard. Her lungs were clear to auscultation bilaterally, with some decreased air movement at the bases. On abdominal exam, she had right upper quadrant abdominal tenderness without a Murphy sign. The abdomen was otherwise soft without rebound tenderness or guarding. Examination of the extremities showed no edema. She appeared to be grossly neurologically intact, with appropriate strength in all extremities.

**Image 1:**
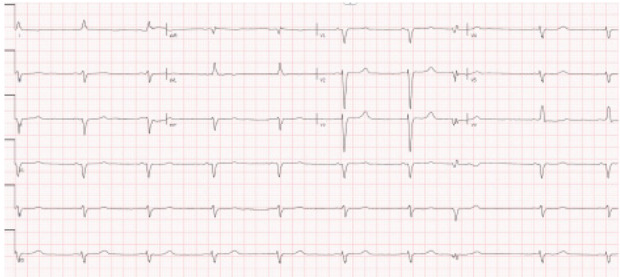
Electrocardiogram of a 70-year-old woman with chest tightness and bradycardia.

**Image 2:**
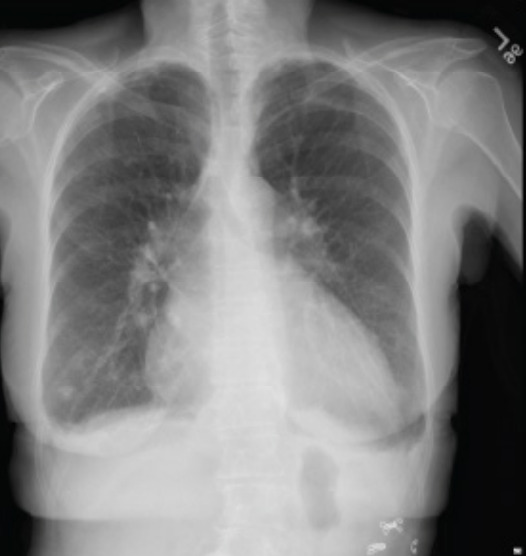
Chest radiograph of a 70-year-old woman with chest tightness and bradycardia.

**Image 3:**
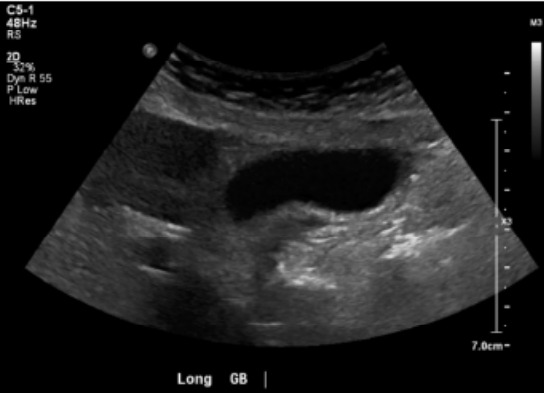
Right upper quadrant ultrasound of a 70-year-old woman with chest tightness and bradycardia.

Laboratory studies were completed (Table 1). The complete blood count was notable for a low hemoglobin and hematocrit. The basic metabolic panel was notable for an elevated blood urea nitrogen (BUN) and creatinine (Cr). Liver function tests were notable for an elevated aspartate aminotransferase, elevated alanine aminotransferase level, and an elevated alkaline phosphatase with normal total bilirubin. Coagulation factors were notable for elevated prothrombin (PT) and activated partial thromboplastin times (aPTT), and an elevated international normalized ratio. An electrocardiogram (ECG) (Image 1), chest radiograph (CXR) (Image 2), and right upper quadrant ultrasound (Image 3) were obtained as well.

The patient remained in no distress. Due to her history of heart failure and current presentation with chest pain, a cardiology consult was obtained while pursuing admission to the hospital. A test was subsequently ordered, and a diagnosis was made.

## CASE DISCUSSION (DR. KLUCHER)

At first glance, this case seems deceptively straightforward. It is tempting to glance at the chief complaint and categorize this patient under the illness script of “older woman with fatigue and chest tightness.” Instinctively, one might begin theorizing whether the urinalysis showing a urinary tract infection or brain-natriuretic peptide indicating a congestive heart failure exacerbation will yield the first abnormal result. After all, how many septuagenarians with similar symptoms do we see every shift?

However, a closer look reveals a more complex scenario: Not only is this older adult with chronic cardiac disease presenting with chest tightness and fatigue, but also with bradycardia and persistent exertional chest pain with shortness of breath. These symptoms have developed over a subacute timeframe. Adding to the puzzle are her constitutional symptoms, generalized weakness and poor appetite, and an array of other complaints, including abdominal discomfort, diarrhea, and muscle aches. The differential diagnosis for this constellation of symptoms is vast, spanning from iatrogenic complications to infectious etiologies and everywhere in between. Her social history of recent immigration and medication nonadherence do not help to simplify this case. Diving into the details is crucial for narrowing down the possibilities and guiding diagnostic testing.

The patient’s physical exam is significant for bradycardia and what may be considered a “soft” blood pressure. She is normothermic. She has a cardiac gallop but otherwise reassuring cardiopulmonary exam including lack of jugular venous distension, basilar crackles, or peripheral edema. Of note, she does have right upper quadrant tenderness.

Her ECG reveals sinus bradycardia with poor R wave progression but without evidence of atrioventricular blocks. There are no prolonged intervals or U waves to suggest electrolyte deficiency as the etiology of her bradycardia. The ECG findings are confirmed by her laboratory values, which demonstrate no significant electrolyte derangements.

These results also steered me away from adrenal insufficiency as an etiology of her fatigue and bradycardia due to the absence of hyponatremia and hyperkalemia. Despite the patient’s cardiac complaints, the troponin level is normal, decreasing immediate concern for an acute injury, such as myocarditis or acute coronary syndrome. Although patients may experience an acute cardiac insult without troponin elevation, a subacute or chronic process with a more insidious onset seems more likely for this patient. She also has a normal white blood cell (WBC) count, although it is worth noting that in the geriatric population an appropriate immune response may not be mounted to acute infection, leading to falsely reassuring normothermia and normal WBC. Thus, infectious processes must remain in play.

She also has increased PT and aPTT. While coagulation tests are not routinely performed for monitoring of direct oral anticoagulation agents, it is possible for dabigatran to cause elevations in PT and aPTT. It may be that our patient is more adherent to her anticoagulant regimen than reported by her family. Gastrointestinal (GI) bleeding from anticoagulation could lead to anemia, abdominal discomfort, diarrhea, and fatigue. Acute GI bleeding could additionally contribute to increased vagal tone and bradycardic episodes but would not explain her persistent bradycardia. Additionally, this patient’s diarrhea is not described as melena or hematochezia, making a GI bleed a less likely explanation for her symptoms.

The question of adherence to dabigatran could indicate that she has been nonadherent to additional medications and had progression or exacerbation of underlying illness such as her heart failure. Alternatively, incorrect dosing or poor understanding of her medications, such as metoprolol, could have led to bradycardia. This would, however, be less likely to cause right upper quadrant pain or transaminitis, and we are missing other signs of beta blocker toxicity such as hypotension or hypoglycemia.

Regarding her imaging, her CXR revealed cardiomegaly and small pleural effusion but without signs of pneumonia or interstitial edema. This is not particularly surprising in a patient with known non-ischemic cardiomyopathy and reduced EF. The right upper quadrant ultrasound was absent of pericholecystic fluid, sludge or stones, or a dilated common bile duct, which when combined with relatively mild transaminitis, makes acute cholecystitis, cholangitis, or primary biliary disorders, including autoimmune disease, less likely.

In the vein of autoimmunity, systemic lupus erythematosus with cardiomyopathy and nephropathy might explain her cardiac symptoms, kidney injury, muscle aches, and fatigue. That said, new-onset systemic lupus erythematosus in a 70-year-old woman would be unexpected as this disease occurs more commonly in younger patients with a peak incidence in patients in their 20s–30s. Furthermore, she lacks photosensitivity and rash, which are the most common presenting symptoms.

Ultimately, I must synthesize her history, symptoms, and workup thus far into a comprehensive picture. When viewing her presentation globally, it seems her exertional chest tightness/pressure and shortness of breath are most likely due to symptomatic bradycardia caused by subacute progression or a new insult to her chronic cardiac disease including heart failure. Her transaminitis and right upper quadrant pain may then be explained by congestive hepatopathy. This could also explain her kidney injury, with a BUN/Cr ratio > 20 suggesting a prerenal azotemia due to decreased renal perfusion from worsening cardiac disease and/or inadequate oral intake (normal BUN/Cr ratio is between 10:1–20:1). Generalized fatigue, poor appetite, and weakness could stem from worsening cardiac disease or as an additional finding of the etiology of her cardiac insult.

Hypothyroidism is common in older women and can contribute to bradycardia, fatigue, and other nonspecific symptoms such as weakness and poor appetite. Hypothyroidism may exacerbate underlying cardiac disease by reducing myocardial contractility and contributing to fluid retention and worsening existing heart failure. I do not yet have a thyroid stimulating hormone available for review; so this could be a reasonable test to consider as I work toward a final diagnosis. It is somewhat reassuring, however, that this patient is not hypothermic or hypotensive, which I might expect to see if she had hypothyroidism severe enough to cause bradycardia and heart failure.

Infiltrative cardiac diseases, such as sarcoidosis or amyloidosis, are important considerations in a patient with bradycardia, fatigue, and heart failure symptoms. These conditions can lead to conduction abnormalities, including bradycardia, as well as progressive myocardial dysfunction due to infiltration or inflammation of the cardiac tissue. These disorders more frequently are associated with diastolic dysfunction and preserved EF, however. This patient also lacks classical findings of these disorders such as perihilar lymphadenopathy in sarcoidosis or hypoalbuminemia and edema suggestive of nephrotic syndrome in amyloidosis.

Infectious causes remain a critical final consideration in this patient’s presentation. Infective endocarditis can lead to conduction abnormalities, heart failure, and constitutional complaints like fatigue and poor appetite. However, the absence of fever and a normal WBC count are less typical, even in older adults who may fail to mount an adequate immune response, and she lacks classic findings of murmur or embolic signs such as Janeway lesions. Other bacterial infections, such as those caused by *Legionella* or *Mycoplasma*, or viral illnesses like influenza and cytomegalovirus, fail to explain the full constellation of cardiac and systemic findings and are less consistent with her timeline and lack of respiratory symptoms.

The patient’s origin from Venezuela introduces important endemic infections into the differential diagnosis. Chronic *Trypanosoma cruzi* infection, the causative agent of Chagas disease, is particularly relevant. Chagas cardiomyopathy is the most common cause of non-ischemic cardiomyopathy in endemic countries, including Venezuela. Over years to decades, it can progress and result in chronic bradycardia, conduction abnormalities, heart failure symptoms, and gastrointestinal complaints such as abdominal pain and diarrhea.^1^ Additionally, although less commonly seen, Chagas hepatopathy could explain her transaminitis and right upper quadrant tenderness independently of congestive effects. Other parasitic diseases, such as schistosomiasis, might also explain hepatosplenic involvement, but her findings are more specific for the cardiac involvement in Chagas disease.

Ultimately, I believe the patient’s symptoms align most closely with Chagas cardiomyopathy, a diagnosis that connects her cardiac history, regional background, and current presentation. While acute Chagas disease is typically diagnosed through blood smear or polymerase chain reaction to detect *T. cruzi* parasitemia, chronic Chagas disease is confirmed through serologic testing to detect antibodies (via enzyme-linked immunosorbent assay [ELISA] or immunofluorescence assay), as parasitemia usually becomes low or undetectable in the chronic phase.^2^ Cardiac manifestations of Chagas disease make chronic infection more likely in this patient. Given her clinical presentation and history, I would order serologic testing for *T. cruzi* to confirm a diagnosis of Chagas cardiomyopathy.

## CASE OUTCOME (DR. DUNN)

Cardiology consult sought to determine the etiology of the patient’s HFrEF. They felt her recent immigration from Venezuela, negative workup from an ischemia standpoint, and partial conduction delay on ECG (ie, QRS duration > 100 milliseconds) were all suspicious for Chagas cardiomyopathy. An infiltrative process, such as sarcoidosis or amyloidosis, was also considered. The cardiology team specifically noted concern for a “cherry-on-top” pattern on the global longitudinal strain analysis of her echocardiogram, which can by suggestive of cardiac amyloidosis and suggested cardiac magnetic resonance imaging (MRI). A *T. cruzi* immunoglobulin G (IgG) antibody ultimately confirmed the diagnosis of Chagas cardiomyopathy.

Her inpatient cardiac MRI demonstrated worsening cardiac function. Her previous EF of 20–25% dropped to 16% in the setting of a severely enlarged left ventricle. Her right ventricle was mildly enlarged with moderately depressed systolic function. A thrombus was additionally identified, adhering to the left ventricular apex. The patient received an automatic implantable cardioverter defibrillator and was discharged after optimization on her guideline-directed medical therapy and anticoagulation.

She had one admission approximately two months after her discharge to a nearby hospital for intermittent chest pain and dyspnea that was felt to be from a viral syndrome, rather than a heart failure exacerbation. Following that admission, she had one additional medication refill from cardiology and was subsequently lost to follow-up.

## RESIDENT DISCUSSION

Chagas disease is caused by a *T. cruzi* infection, which is a protozoan parasite transmitted by the triatomine or reduviid “kissing” bug. The fecal matter of the bug contains a parasite that inoculates the host through bite wounds or intact mucosal membranes. Vertical transmission is also a possibility and can present asymptomatic at birth. Finally, infection can also occur via infected organ transplantation or blood transfusion.^3^ The disease is named after Dr. Carlos Chagas, a Brazilian physician who first identified the causative agent and insect vector.

### Epidemiology

Chagas disease is endemic in 21 continental Latin American countries with an estimated eight million people infected in the Americas, and an estimated 280,000 infected individuals living in the United States. As these bugs hide in thatch roofing and unfinished housing, infection is prevalent in rural areas. It is important to note that short-term travel to these endemic countries is not a risk factor to infection as it requires long-term stay in these rural areas where people are repeatedly bitten or exposed to the triatomine bugs.^3^

### Pathophysiology

Infection starts with an asymptomatic incubation period of 1–2 weeks. The subsequent acute phase lasts 8–12 weeks and can also be asymptomatic or have non-specific symptoms such as fever, malaise, or anorexia. About 1% of acute phase patients will have complications such as a pericardial effusion or meningoencephalitis. Once patients enter the chronic phase, after 12 weeks, they can begin to develop heart or GI disease.^4^

Chagas cardiomyopathy has four major manifestations: heart failure; cardiac arrhythmias; thromboembolism; and chest pain syndrome. The parasite’s presence in the body causes a chronic inflammatory state. In the heart, this leads to fibrosis of the ventricular wall, resulting in dilated cardiomyopathy, valvular regurgitation, and ultimately biventricular failure. Fibrosis can additionally occur in the conduction system, leading to atrioventricular blocks, ventricular rhythms, and other dysrhythmias.^5^ About 55–65% of deaths in patients with Chagas disease are secondary to sudden cardiac death from a dysrhythmia. The chronic inflammatory state also increases the risk of thromboembolism and strokes, with thrombi developing within the dilated cardiac chambers of the heart.^6^ Finally, patients can develop a chest pain syndrome that may mimic angina but is suspected to be due to myocardial microvascular abnormalities from the cardiac remodeling from the parasite’s presence.

### Diagnostics

In the acute phase of Chagas, a diagnosis is made by visualizing trypomastigotes in blood using microscopy. Unfortunately, outside existing screening programs, most patients are diagnosed after the relatively short acute phase. Chronic disease may be identified with IgG antibody testing for *T, cruzi*. These antibodies develop 2–8 weeks after infection. Due to antigenic diversity of *T. cruzi*, it is recommended to obtain two serologic tests to achieve adequate sensitivity. Immunoglobin G antibody testing using an ELISA methodology has a 99% sensitivity and 98% specificity in detection.^7^ Patients at risk of chronic disease should be assessed for potential cardiac complications, including an ECG for conduction abnormalities, CXR for cardiomegaly, and an echocardiogram if there is concern for valvular regurgitation, thrombus, and/or wall motion abnormalities.^8^

### Treatment

Acute Chagas disease is managed with antitrypanosomal medications. First-line treatment for adults is benznidazole, usually at 5 mg/k daily, administered in two divided doses for 60 days. It is worth noting that use of benznidazole for this purpose in patients > 12 years of age is off label but generally better tolerated than the primary alternative, nifurtimox. The role of antitrypanosomal treatment outside acute, congenital, or reactivated cases of *T. cruzi* is less clear.^9^

Historically, treatment of chronic Chagas cardiomyopathy has depended on the severity of the heart failure symptoms. A patient must meet the criteria of the American Heart Association Class A or B or the New York Heart Association Class 0 or 1 to receive antitrypanosomal therapy and find benefit. At this stage, this patient does not have evidence of structural heart disease or heart failure symptoms and, as suggested by a 2015 randomized multicenter trial of benznidazole, will have a reduced incidence of death from cardiovascular events from Chagas cardiomyopathy.^10^ Antitrypanosomal therapy historically has not been thought to have mortality benefit after progression to symptomatic heart failure, although some authors have recently posited that a greater number of patients may benefit.^8^,^11^

All heart failure patients should be started on guideline-directed medical therapy. Patients should have annual ECGs and cardiology visits. If patients have a normal left ventricular EF, they can usually receive an echocardiogram every 3–5 years, but those with an EF < 50% should receive annual studies. Clinicians can also use the Rassi score to help stratify mortality risk in patients with Chagas disease. The Rassi score is a prognostic assessment that incorporates factors such as the New York Heart Association class, presence of cardiomegaly on CXR, and segmental or global left ventricular systolic dysfunction. Severe cases may require cardiac transplantation. Evidence shows that cardiac transplant recipients have a survival benefit and that reactivation of *T. cruzi* is a rare cause of death.^8^

## FINAL DIAGNOSIS

Chagas cardiomyopathy

**KEY POINTS**

1. Have a high index of suspicion for Chagas disease in a patient with heart failure and a history of residence in rural areas of continental Latin America.
2. Acute Chagas disease and some chronic Chagas cardiomyopathy patients can be treated with antitrypanasomal therapy.
3. As a patient’s Chagas cardiomyopathy progresses, their therapy is consistent with guideline-directed medical therapy including heart transplant, which has been shown to have a mortality benefit.

## Figures and Tables

**Table 1:** Initial laboratory results of a 70-year-old woman with chest tightness and bradycardia.

Test	Patient value	1 month prior	Normal value
Complete Blood Count			
White blood cell count	4.6 K/mcL	5.28 K/mcL	4.0–10.0 K/mcL
Hemoglobin	9.7 g/dL	10.6 g/dL	12.0–14.7 g/dL
Hematocrit	31.6%	34.9%	36.0–45.0%
Platelets	230 K/mcL	250 K/mcL	166–362 K/mcL
Serum Chemistries			
Sodium	138 mmol/L	135 mmol/L	136–145 mmol/L
Potassium	5.0 mmol/L	4.8 mmol/L	3.5–5.1 mmol/L
Chloride	106 mmol/L	96 mmol/L	98–107 mmol/L
Bicarbonate	21 mmol/L	26 mmol/L	21–30 mmol/L
Blood urea nitrogen	35 mg/dL	26 mg/dL	7–17 mg/dL
Creatinine	1.7 mg/dL	1.6 mg/L	0.52–1.04 mg/dL
Glucose	131 mg/dL	100 mg/L	70–99 mg/dL
Anion gap	10 mmol/L	13 mmol/L	
Calcium	9.2 mg/dL	9.5 mg/dL	8.6–10.2 mg/dL
Magnesium	2.3 mg/dL	2.4 mg/dL	1.6–2.6 mg/dL
Phosphorus	4.7 mg/dL	- not reported -	2.8–4.5 mg/dL
Total protein	7.9 g/dL	8.0 g/dL	6.0–8.3 g/dL
Albumin	4.2 g/dL	4.1 g/dL	3.4–5.4 g/dL
Hepatic Studies			
Aspartate aminotransferase	77 u/L	- not reported -	14–36 u/L
Alanine aminotransferase	45 u/L	48 u/L	0–34 u/L
Alkaline phosphatase	147 u/L	148 u/L	38–126 u/L
Total bilirubin	1.0 mg/dL	0.8 mg/dL	0.3–1.2 mg/dL
Cardiac Studies			
Troponin I	0.03 ng/mL		< 0.06 ng/mL
Troponin T – high sensitivity		51 ng/L (1^st^) 47 ng/L (2^nd^)	< 14 ng/L

*dL*, deciliter; *g*, gram; *K*, thousand; *L*, liter; *mcL*, microliter; *mmol*, millimole; *mg*, milligram; *mL*, milliliter; *ng*, nanogram; *u*, units.
